# Prevalence and risk factors for active convulsive epilepsy in rural northeast South Africa

**DOI:** 10.1016/j.eplepsyres.2014.01.004

**Published:** 2014-05

**Authors:** Ryan G. Wagner, Anthony K. Ngugi, Rhian Twine, Christian Bottomley, Gathoni Kamuyu, F. Xavier Gómez-Olivé, Myles D. Connor, Mark A. Collinson, Kathleen Kahn, Stephen Tollman, Charles R. Newton

**Affiliations:** a Studies of Epidemiology of Epilepsy in Demographic Surveillance Systems (SEEDS)–INDEPTH Network, Accra, Ghana; b MRC/Wits Rural Public Health & Health Transitions Research Unit (Agincourt), School of Public Health, Faculty of Health Sciences, University of the Witwatersrand, Johannesburg, South Africa; c Division of Epidemiology and Global Health, Department of Public Health and Clinical Medicine, Umeå University, Umeå, Sweden; d KEMRI/Wellcome Trust Research Programme, Centre for Geographic Medicine Research–Coast, Kilifi, Kenya; e Research Support Unit, Faculty of Health Sciences, Aga Khan University- East Africa, Nairobi, Kenya; f Department of Infectious Disease Epidemiology, Faculty of Epidemiology and Population Health, London School of Hygiene and Tropical Medicine, London, United Kingdom; g MRC Tropical Epidemiology Group, Faculty of Epidemiology and Population Health, London School of Hygiene and Tropical Medicine, London, United Kingdom; h Borders General Hospital, Melrose, United Kingdom; i Neurosciences Unit, UCL Institute of Child Health, London, United Kingdom; j Clinical Research Unit, London School of Hygiene and Tropical Medicine, London, United Kingdom; k Department of Psychiatry, University of Oxford, Oxford, United Kingdom

**Keywords:** Epilepsy, Prevalence, Case-control, Risk factors, Population-based

## Abstract

**Rationale:**

Epilepsy is among the most common neurological disorders worldwide. However, there are few large, population-based studies of the prevalence and risk factors for epilepsy in southern Africa.

**Methods:**

From August 2008 to February 2009, as part of a multi-site study, we undertook a three-stage, population-based study, embedded within the Agincourt health and socio-demographic surveillance system, to estimate the prevalence and identify risk factors of active convulsive epilepsy (ACE) in a rural South African population.

**Results:**

The crude prevalence of ACE, after adjusting for non-response and the sensitivity of the screening method, was 7.0/1,000 individuals (95%CI 6.4–7.6) with significant geographic heterogeneity across the study area. Being male (OR = 2.3; 95%CI 1.6–3.2), family history of seizures (OR = 4.0; 95%CI 2.0–8.1), a sibling with seizures (OR = 7.0; 95%CI 1.6–31.7), problems after delivery (OR = 5.9; 95%CI 1.2–24.6), and history of snoring (OR = 6.5; 95%CI 4.5–9.5) were significantly associated with ACE. For children, their mother's exposure to some formal schooling was protective (OR = 0.30; 95%CI 0.11–0.84) after controlling for age and sex. Human immunodeficiency virus was not found to be associated with ACE.

**Conclusions:**

ACE is less frequent in this part of rural South Africa than other parts of sub-Saharan Africa. Improving obstetric services could prevent epilepsy. The relationship between snoring and ACE requires further investigation, as does the relative contribution of genetic and environmental factors to examine the increased risk in those with a family history of epilepsy.

## Highlights

• Epilepsy is prevalent in rural South Africa, but less than other parts of Africa.
• Most epilepsy starts in childhood.
• Poor obstetric history and snoring were associated with active convulsive epilepsy.
• HIV and parasitic infection were not associated with active convulsive epilepsy.

## Introduction

Epilepsy is one of the most common neurological disorders in the world, affecting about 69 million people worldwide, with 90 percent living in low- and middle-income countries (LMICs) (Ngugi et al., 2010). It contributes nearly one percent to the global burden of disease (Murray et al., 2012), and 20 percent of the global burden of epilepsy is in Africa (World Health Organization, 2004).

While these figures suggest a large burden of epilepsy in Africa, they are derived from a limited number of studies that employ different case definitions and methodologies. Studies suggest that utilizing hospital records in LMICs to detect epilepsy under-estimates the prevalence by at least 80 percent due to limited health care utilization by people with epilepsy (PWE) in these settings (Osuntokun et al., 1987). Thus, population-based surveys are frequently used to estimate prevalence, though not without limitations, including the absence of well-demarcated populations and vital statistics registries. This limitation, coupled with the lack of trained medical personnel available to make the diagnosis of epilepsy, makes estimating the burden of epilepsy in sub-Saharan Africa unusually challenging.

A recent systematic review and meta-analysis highlighted the significant variation in the prevalence of epilepsy between high-income countries and LMICs, with a higher prevalence in LMICs, especially in rural settings (Ngugi et al., 2010). The authors suggest that study size and the economic development level of the study country largely explain the heterogeneity, although increases in obstetric injury, head injuries, and infections and infestations of the central nervous system (CNS) (Newton & Garcia, 2012), such as toxoplasmosis and toxocara (Wagner & Newton, 2009), malaria (Carter et al., 2004) and human immunodeficiency virus (HIV) are thought to contribute (Ngugi et al., 2010), but there is little data from South Africa where the prevalence of HIV is very high.

As part of a multi-centre study on the epidemiology of epilepsy in demographic sites (SEEDS) (Ngugi et al., 2013), we conducted a three-stage, population-based survey and a case-control study to determine the prevalence of and risk factors for active convulsive epilepsy (ACE) in rural South Africa. In particular, we were interested in examining the risk factors for ACE in a non-malaria endemic area, particularly HIV since it has a high prevalence in South Africa.

## Methodology

### Population and study area

**Figure 1:**
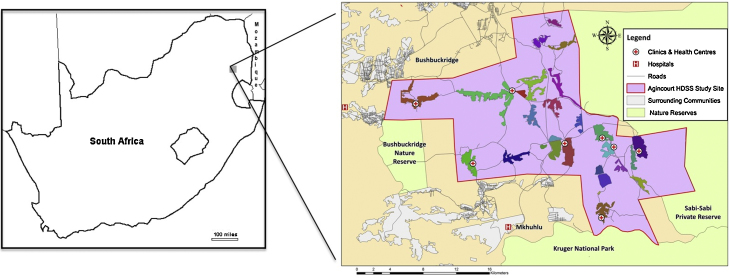
Map of South Africa, Bushbuckridge Municipality, and the Agincourt sub-district and health and socio-demographic surveillance system, 2008.

The study was conducted in the rural Agincourt health sub-district, in which the Agincourt health and socio-demographic surveillance system (HDSS) operates and is located 500 kilometers northeast of Johannesburg (Fig. 1). The Agincourt HDSS was established in 1992 as a research platform to inform health and development policy through evidence-based research (Kahn et al., 2012). The population has been enumerated through an annual census update, following baseline measurement in 1992 and captures vital statistics including births, deaths, and in- and out-migrations.

In 2008, the population was 83,121 individuals in 15,841 households and 25 villages on 420 km^2^ of semi-arid scrubland. The site forms part of a former *bantustan*, or ethnic ‘homeland’ implemented during the Apartheid era. The population is mainly Xi-Tsonga speaking, with one-third (32.8%) originally from Mozambique.

The leading causes of death ascertained through the Agincourt HDSS are HIV/AIDS, cardiovascular disease and trauma (road traffic accidents, assaults) (Tollman et al., 2008). Six government clinics, one larger government health centre, and one public-private community health centre, with its main focus being HIV and tuberculosis, provide primary health care services for the population. Referrals are to three government district hospitals located 25 to 55 km from the sub-district.

### Ethics

Ethical clearance for the study was received from the Human Research Ethics Committee of the University of the Witwatersrand, Johannesburg, South Africa (Clearance number: M080455) and the Mpumalanga Province Department of Health's Research and Ethics committee. Written informed consent was sought from each participant in the study. Parental or guardian informed consent was sought in the case of children or patients with cognitive impairment, with verbal assent being sought from children and cognitively impaired patients.

### Case definition of active convulsive epilepsy (ACE)

We identified patients with Active convulsive epilepsy (ACE) since convulsive epilepsies are associated with increased morbidity, mortality (Diop et al., 2005) and greater social stigma than non-convulsive epilepsies (World Health Organization, 2005). ACE was defined as having two or more unprovoked convulsive seizures occurring more than 24 hours apart, with at least one seizure occurring in the 12 months preceding the study or currently taking anti-epileptic drugs (AEDs) (Edwards et al., 2008).

### Procedures

Between August and November 2008, two questions (‘Do you have fits or has someone ever told you that you have fits?’ and ‘Do you experience episodes in which you legs or arms have jerking movements or fall to the ground and lose consciousness?’), used previously in a similar study (Edwards et al., 2008), were administered by census fieldworkers to a senior member of each household (*n* = 15,841) on behalf of each member of the household. The questions had been piloted previously in the local language (Xi-Tsonga) at a district hospital and had a high specificity (100%) and sensitivity (98%). The questions were included in the 2008 annual Agincourt HDSS census update round. The two questions within the census update constituted Stage I of the study and sought to identify all people who had experienced a seizure prior to 1 August 2008 (the prevalence day).

In Stage II (August–December 2008), trained fieldworkers visited all individuals who responded ‘yes’ to either of the two questions in Stage I and asked a detailed questionnaire, based on previous studies (Placencia et al., 1992), about the characteristics and history of the seizures. Fieldworkers were specifically trained by the study managers (RGW, AKN) and study neurologist (CRN) on the administration of this questionnaire. Individuals who had experienced at least two seizures in their lifetime and at least one seizure with abnormal movement (aimed at identifying convulsive epilepsy) in the preceding 12 months or currently taking AEDs were invited for further assessment in the epilepsy clinic within the following week (Stage III).

In Stage III (September 2008–February 2009), socio-demographic information was obtained and a specially trained nurse made the diagnosis of ACE, based on a positive clinical history provided by the patient's caregiver or family member. A clinical examination was completed to identify neurological abnormalities and physical co-morbidities. Blood was drawn from each patient for serological testing. A neurologist (CRN) confirmed the diagnosis of epilepsy by reviewing all of the patient records and examining selected patients.

### Population sample

In addition to identifying cases of epilepsy through the population screen (Stage I), a random sample of 4,500 individuals (all ages and both sexes), from the Agincourt HDSS, were screened with the Stage II tool. This population sample simulated the traditional two-stage epilepsy prevalence study design (Placencia et al., 1992) and was used to evaluate the three-stage methodology of this study. Those who screened positive were referred for assessment in Stage III and included as cases in the case-control study.

Furthermore, individuals who were referred to the epilepsy clinic by sources (clinic staff and community leaders) other than the three-stage study were clinically assessed and those found to have ACE were included as cases in the case-control study.

### Selection of controls for case control study

Controls were selected randomly from those that screened negative for epilepsy in the population sample and frequency matched to cases diagnosed with ACE during Stage III of the study (on age-bands 0–5, 6–12, 13–18, 19–28, 29–49, and 50+ years). If the selected control was not found after three attempted visits, another control was selected randomly as a replacement. For each control selected, a clinical history and blood sample were taken and a clinical examination performed.

### Serological testing

Blood from cases and controls was collected in Stage III of the study and exposure to *Toxocara canis* excretory-secretory antigen with a sensitivity of 97.1% and specificity of 78.6% (Noordin et al., 2005), toxoplasmosis (Toxoplasma IgG ELISA Kit, Genesis Diagnostics Ltd, Product Code GD80 (Liesenfeld et al., 1996)), and *Plasmodium falciparum* IgG antibodies tested by ELISA was examined. Exposure to the larval stage (cysticercosis) and adult stage (taeniasis) of the parasite *T. solium* in the patients only, given the absence of cysticersosis reported by the local radiologists. HIV antibodies (Vironostika HIV Uni-form II Ag/Ab Kit) were also measured after written informed consent was received from each individual or their legal guardian.

### Statistical analysis

Data were double entered into a mySQL database (OracleCorp, Redwood Shores, CA, USA). All analyses were conducted using Stata 10 (StataCorp, College Station, TX, USA).

We adjusted for attrition between the stages of the survey using a multiple imputation method. The figures were also adjusted for the sensitivity of the three-stage method, previously calculated in a separate study (Ngugi et al., 2012) (Table 1). We report adjusted prevalence by age, sex, and village of residence. We also present prevalence estimates standardized to the 2000 US population (US Census Bureau, 2000) to allow for global comparison and the median and interquartile range of age of onset of ACE. Finally, we report the adjusted prevalence figures from the population sample of 4,500 individuals.

**Table 1:** Crude and adjusted prevalence of ACE by age and sex, Agincourt sub-district, South Africa 2008.

Age band	Cases of ACE	Crude prevalence	95% Confidence interval	Adjusted prevalence	95% Confidence interval
0 to 5 years	10	0.87	0.33–1.41	2.18	0.88–3.48
6 to 12 years	29	2.23	1.42–3.03	4.79	3.09–6.52
13 to 18 years	34	2.76	1.83–3.68	6.38	4.32–8.42
19 to 28 years	47	2.65	1.89–3.40	6.23	4.47–7.98
29 to 49 years	87	4.70	3.71–5.69	10.95	8.74–13.13
50+ years	38	3.94	2.68–5.19	9.55	6.69–12.41
Male	129	3.24	2.68–3.80	7.43	5.53–7.82
Female	116	2.69	2.20–3.18	6.50	4.53–6.54

### Factors associated with active convulsive epilepsy

In the case-control study, we performed analysis on 20 socio-demographic and clinical variables and four serological variables (Table 3). We developed models using logistic regression and in each model included a variable of interest, adjusted for age and sex. In patients >18 years of age, variables on whether alcohol was consumed and period of consumption were analyzed. Three additional variables were analyzed for patients <18 years old: mother's marital status, whether the mother experienced some formal education, and her level of education.

**Table 3:** Multivariate analysis of possible risk factors associated with active convulsive epilepsy, Agincourt sub-district, 2008.

Variable of interest	Controls (*n* = 260)	Cases (*n* = 292)	Odds ratio (95% CI)^a^	*p*-value
Sex				
Female	176 (67)	142 (49)	1	
Male	85 (33)	150 (51)	2.27 (1.60–3.23)	<0.001
Family history of non-febrile seizures				
Someone in family with seizures	11 (4)	41 (14)	4.01 (1.99–8.09)	<0.001
Someone in family with past history of seizure	0 (0)	12 (4)	b	<0.001
Mother with Seizures	0 (0)	3 (1)	b	0.146
Father with seizures	1 (0)	2 (1)	1.87 (0.16–21.7)	0.615
Sibling with seizure	2 (1)	14 (5)	7.05 (1.57–31.68)	0.011
Location of birth				
Hospital	9 (5)	17 (6)	1	
Clinic	109 (62)	144 (54)	0.68 (0.29–1.60)	0.379
Home	57 (32)	97 (36)	0.66 (0.26–1.66)	0.379
Unknown	2 (1)	8 (3)	1.58 (0.267–9.38)	0.612
Adverse perinatal events				
Normal delivery	163 (96)	246 (93)	1	
Abnormal delivery	7 (4)	19 (7)	2.05 (0.81–5.15)	0.128
No problems after delivery	174 (99)	249 (94)	1	
Problems after delivery	2 (1)	14 (5)	5.93 (1.18–24.58)	0.030
History of febrile seizures	0 (0)	3 (1)	b	0.101
History of head trauma				
No history of head injury	245 (94)	260 (89)	1	
History of head injury	16 (6)	31 (11)	1.57 (0.82–2.99)	0.170
Loss of consciousness with the head injury	3 (19)	20 (65)	2.69 (0.68–10.61)	0.157
Sociodemographic and clinical characteristics				
Abnormal skull shape	0 (0)	23 (8)	b	<0.001
History of eating cassava	154 (59)	190 (66)	1.31 (0.92–1.88)	0.137
History of eating pork	69 (26)	59 (20)	0.70 (0.47–1.04)	0.078
History of eating soil	19 (7)	18 (6)	1.05 (0.53–2.08)	0.878
History of dogs in dwelling	52 (20)	68 (23)	1.17 (0.77–1.77)	0.457
History of cats in dwelling	22 (8)	16 (6)	0.64 (0.33–1.27)	0.202
History of snoring (at least 3x/week)	51 (20)	187 (64)	6.51 (4.45–9.53)	<0.001
Serological variables				
Positive for malarial antibodies	71 (33)	78 (39)	1.16 (0.76–1.78)	0.485
Positive for HIV antibodies	49 (23)	36 (18)	0.73 (0.44–1.21)	0.226
Toxocara	77 (36)	73 (37)	1.17 (0.78–1.79)	0.444
Toxoplasmosis	22 (10)	27 (14)	1.20 (0.64–2.25)	0.580
*n* (%)				

a Odd Ratios adjusted for age and sex.

b Indicates that an Odds ratio could not be calculated due to zero exposure in the control population.

## Results

**Figure 2:**
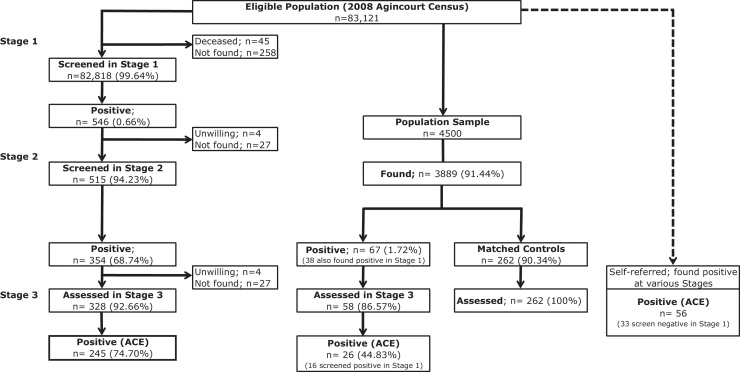
Study design schema and numbers of individuals at each stage, 2008.

Participation in the three-stages of the survey is displayed in Fig. 2.

### Prevalence

We identified 245 cases of ACE in the three-stage study. The unadjusted prevalence of ACE was 3.0/1,000 individuals (95%CI: 2.6–3.3) within the three-stage study. Adjusting for attrition and the sensitivity of the three-stage methodology (48.6%), the adjusted crude prevalence was 7.0/1,000 (95%CI: 6.4–7.6). The sensitivity of 48.6% for the three-stage methodology was derived from a validation study performed in Kenya using clinical assessments as the gold standard (Ngugi et al., 2012). The prevalence, standardized to the age distribution of the US population in 2000 and adjusted for attrition and sensitivity of the three-stage method, was 8.1/1,000 individuals (95%CI: 7.5–8.7).

Within the population sample of 3,889 (611 not found due to permanent out-migration from the study area) the crude prevalence was 6.7/1,000 individuals (95%CI: 4.1–9.3), while the adjusted prevalence (adjusting for attrition and sensitivity (76.7%) of the two-stage method against the gold standard (Ngugi et al., 2012)) was 9.8/1,000 individuals (95% CI: 6.9–13.4).

The adjusted prevalence of ACE by village ranged from 1.8–15.0/1,000 individuals (Table 2) and this variation was statistically significant (*p* = 0.05), although the response rate for Stage 1 and Stage 2 did not vary between villages.

**Table 2:** Adjusted prevalence of active convulsive epilepsy by village of residence, Agincourt sub-district, 2008.

Village^a^	Cases of ACE	Adjusted prevalence	95% Confidence interval
A	7	7.17	5.36–9.40
B	3	2.77	1.26–5.25
C	20	6.21	4.30–8.67
D	22	10.92	7.78–14.90
E	6	6.31	3.74–9.96
F	11	5.46	3.34–8.42
G	9	7.24	4.22–11.57
H	13	4.67	2.99–6.84
I	1	6.84	4.68–9.65
J	2	5.1	3.27–7.58
K	11	7.39	5.49–9.73
L	3	3.02	1.12–6.21
M	6	3.02	1.21–6.21
N	1	6.77	4.30–10.14
O	6	11.41	7.07–17.38
P	10	8.45	5.53–12.35
Q	7	10.04	7.25–13.55
R	17	14.98	10.06–21.45
S	18	6.64	2.67–13.62
T	11	3.03	0.06–8.83
U	16	9.91	5.67–16.04
V	10	7.2	5.24–9.66
W	4	7.07	4.05–11.46
X	10	9.96	6.17–15.18
Y	21	1.77	0.21–6.28
Overall	245	7.01	6.23–7.78

a Villages have been anonymized due to small village size and confidentiality of individuals with ACE.

### Age of onset of epilepsy

**Figure 3:**
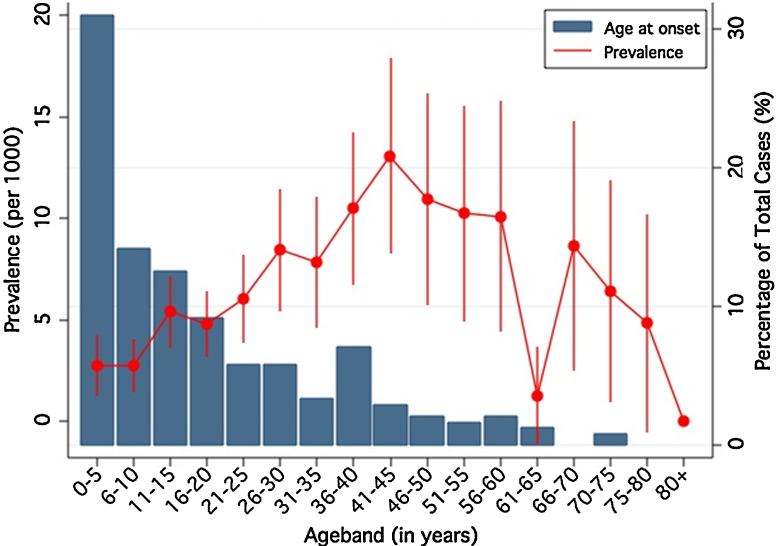
Age of onset (bars) and prevalence with 95% confidence intervals (by 5-year age bands) of active convulsive epilepsy, Agincourt sub-district, 2008.

239 individuals reported age of onset of their seizures. The distribution of age of onset was left-skewed with 7.1% (*n* = 17) experiencing seizures within the first year of life (Fig. 3) and 30.8% (*n* = 74) before 5 years. The median age at onset was 12 years interquartile range was 0–23 years.

### Factors associated with epilepsy

Of the 311 cases of ACE identified (245 from the three-stage survey, 10 additional cases from the population sample, and 56 cases referred from other sources), 292 cases (94%) were matched to 260 controls. The matching was not 1:1 due to a high proportion of refusals in the controls.

### Univariate analysis

Within the univariate analysis, male sex, family history of seizures, a sibling with seizures, problems after delivery, loss of consciousness during head injury and history of snoring at least three nights per week were associated with ACE. ACE was not associated with exposure to any of the parasites tested and none of the cases had antibodies to *T. solium*.

For individuals >18 years, neither alcohol consumption nor length of alcohol consumption was associated with ACE.

For individuals <18 years, having a mother with some history of formal education reduced her child's risk of ACE (OR = 0.34; 95%CI 0.12–0.94), while history of snoring at least three nights per week (OR = 6.51; 95%CI 3.25–13.06) was associated with an increased prevalence of ACE. Neither the mother's level of formal education nor mother's marital status were significantly associated with ACE.

### Multivariate modeling

After controlling for age and sex, a family member with seizures, a sibling with seizures, problems after delivery, and history of snoring at least three nights per week were associated with ACE (Table 3).

Additionally, for individuals <18 years, a mother with formal education (OR = 0.30; 95%CI 0.11–0.84) was found to be protective.

While head injury was not associated with ACE in the multivariate model, males were twice as likely to experience head injury (OR = 2.01; 95%CI 1.06–3.88) when compared with females.

## Discussion

From this community-based research in rural northeast South Africa, we found the prevalence of epilepsy in a rigorous three-stage study to be lower than that reported elsewhere in sub-Saharan Africa (SSA). After adjusting for the sensitivity of the screening tool and attrition in the three stage survey, our findings remain lower than the 12.7/1,000 cases of active epilepsy reported in a meta-analysis of studies in rural areas of LMICs (Ngugi et al., 2010) and other SEEDS sites in SSA (Ngugi et al., 2013). This could be explained by differences in methodology or risk factors (Ngugi et al., 2013). ACE may only constitute 25% of all epilepsies (Cockerell et al., 1995, Edwards et al., 2008). In high-income countries, only half of the epilepsies are convulsive and about half of patients with convulsive epilepsy experienced a seizure within the last year (Cockerell et al., 1995). Therefore, the real prevalence of active epilepsy may well be higher than the adjusted, age-standardized prevalence found in this study (8.1/1,000). Furthermore, the increased mortality associated with epilepsy in Africa (Diop et al., 2005) and the higher rates of spontaneous remission (Newton & Garcia, 2012) may lower the prevalence of active epilepsy. We suspect the lower unadjusted prevalence figure recorded in the three-stage study may also be the result of social stigma. A recent study from Kenya found that false negative individuals in a similar three-stage study reported higher stigma scores on a validated stigma scale than those positively identified in Stage I (Ngugi et al., 2012). While stigma levels are likely to vary depending on social perceptions of epilepsy, further research is needed to define the extent of under-reporting.

### Prevalence by sex

Epilepsy studies from SSA report varying levels of prevalence by sex (Birbeck & Kalichi, 2004). Our data suggest that males are more likely to develop epilepsy than females during the first two decades of life, perhaps due to risk factor patterns discussed below.

### Age of onset

These findings from rural South Africa replicate those found in Tanzania and Ethiopia as well as the other SEEDS sites (Kenya, Tanzania, Uganda and Ghana), which suggest that the highest incidence of epilepsy occurs in the first decade of life (Rwiza et al., 2005, Tekle-Haimanot et al., 1997, Ngugi et al., 2013). We found the onset of epilepsy to be greatest in the first year of life, which is supported by our case-control data in which problems after delivery were associated with ACE. Underlying genetic or congenital abnormalities could possibly be important causes of epilepsy in this community. The second peak of onset during adolescence (10–15 years) may be indicative of environmental, infectious and traumatic risk factors. The increased onset during the fourth decade of life (30–39 years) may reflect the bimodal distribution of epilepsy seen in high-income countries (World Health Organization, 2005). South Africa is experiencing increasing levels of cardiovascular disease (Mayosi et al., 2009), which may increase the incidence of epilepsy later in life, although this tends to occur at younger age in populations undergoing epidemiological transition than in high income populations (Connor et al., 2007). Further research is needed to determine the underlying causes of symptomatic epilepsy in rural South Africa.

### Prevalence by village of residence

There are a number of possible reasons for the inter-village heterogeneity of the prevalence of ACE across the Agincourt sub-district. While attrition of respondents between stages did not vary significantly by village, it is possible that under-reporting of convulsions in Stage I (suggested by 10 false-negatives in the population sample) contributed to the observed village-level heterogeneity. Differences in response rates and awareness of epilepsy have been highlighted as possible causes of heterogeneity in small areas (Edwards et al., 2008).

Genetic abnormalities, exposure to a particular environmental risk factor, or access to health facilities (in terms of perinatal care) may also contribute to the heterogeneity, as we identified several households with more than one person with epilepsy. Ultimately, however, it is possible that the heterogeneity is due to combination of factors or may reflect the actual distribution of epilepsy in a rural South African setting.

### Factors associated with ACE

The factors associated with ACE were different in Agincourt compared to other SEEDS sites (Ngugi et al., 2013).

#### Sex

We found a higher prevalence in males than in females, which replicates other studies from SSA (Preux & Druet-Cabanac, 2005). Being male may put an individual at greater risk for known risk factors, such as head injury. Within this study, males with epilepsy were twice as likely to have experienced a head injury than females with epilepsy. Road traffic accidents and assault–both common causes of traumatic brain injury and potential risk factors for epilepsy–were found to be leading causes of death in individuals aged 15–49 years within the Agincourt sub-district (Tollman et al., 2008). Furthermore, it has also been reported that females are more likely to hide their epilepsy (Preux & Druet-Cabanac, 2005), which may contribute to the higher prevalence observed in males.

#### Family history of seizures

A family history of seizures was shown to be a significant risk factor for developing convulsive epilepsy in this study, as in other studies from SSA (Edwards et al., 2008, Matuja et al., 2001, Nsengiyumva et al., 2003). Small numbers in our study limited exploration of relationships between family members experiencing seizures; however, this study did find that having a sibling with seizures is a significant risk factor for epilepsy. Further research could explore these relationships and the underlying etiology to assess the balance between genetic, social and environmental exposures. The large and closely related family samples in much of rural SSA could support expanded research into genetic risk factors for ACE (Farnarier & Genton, 1996).

#### Adverse perinatal events

Problems after delivery were found to be a risk factor for developing epilepsy, while problems during delivery do not appear to be a risk factor. Studies from Tanzania and Burundi found the risk of developing ACE to be 4.5 and 1.9 times greater, respectively, after perinatal complications (Matuja et al., 2001, Nsengiyumva et al., 2003). In the current study, at least 60% of births occurred at clinics or hospitals. In this regard, rural South Africa may differ from the rest of SSA where most children are born at home without professional support (Diop et al., 2003).

#### Snoring

Snoring was included to detect sleep disturbance in people with epilepsy, as documented elsewhere (Van Golde et al., 2011). We found that snoring was significantly associated with ACE, though snoring was not associated with body mass index, age or sex. Snoring is a symptom in obstructive sleep apnea (OSA) (American Sleep Disorders Association, 2001) and a number of studies have found a high prevalence of sleep disorders, including OSA, in people with epilepsy (Beran et al., 1999, Marlow et al., 2000, Marlow et al., 2008, Kader et al., 2010). While snoring was associated with ACE in this study, we cannot extrapolate any temporal relationship or etiological association between snoring and seizures. Furthermore, snoring may be a risk factors or a consequence of a co-morbid condition, such as sleep apnea. Additional research is warranted to better understand this unusual finding.

#### Mother's experience with formal education

In individuals <18 years of age, having a mother who attended some formal education was found to be a protective factor, which differs from earlier research in LMICs (Hackett et al., 1997, Mung’ala-Odera et al., 2008). Education is often linked with higher socio-economic status, which in turn can be linked with better access to healthcare. Further research to establish the temporality and causal pathway of a mother's education as a protective factor for her child developing ACE is warranted.

### Infections known to be associated with ACE

Toxocara, toxoplasmosis, HIV and malaria were not significantly associated with ACE in this study, which differs from other SEEDS sites (Ngugi et al., 2013). None of the patients with ACE had evidence of exposure to neurocysticercosis, an established risk factor for epilepsy in other parts of Africa. These results differ markedly from the Eastern Cape province of South Africa, where an estimated 34,662 cases (95% CI: 17,167–54,068) of epilepsy related to neurocysticercosis occurred in 2004 (Carabin et al., 2006). This difference is likely the result of very little free-range pig farming within the Agincourt sub-district and regionally, coupled with some local religions forbidding consumption of pork.

#### Human immunodeficiency virus (HIV)

Surprisingly, we did not find HIV infection to be a risk factor for ACE, even though the prevalence of HIV infection in this area is high (18% of cases and 23% of controls in this study) (Gómez-Olivé et al., 2013). While HIV was not shown to be a risk factor for ACE, studies elsewhere report new-onset seizures to be associated with HIV infection and subsequent opportunistic infections (Satishchandra and Sinha, 2008). HIV was furthermore not associated with adult onset epilepsy nor was toxoplasmosis associated with HIV in this study. Due to the cross-sectional design of this study, individuals at risk for epilepsy, because of an opportunistic infection due to HIV, may be missed due to early mortality. More research on interactions between HIV and epilepsy is indicated, particularly in the absence of opportunistic cerebral infections.

## Conclusion

This study, the largest population-based epilepsy study in rural South Africa to-date, found convulsive epilepsy to be less frequent in this area than in other parts of SSA, with significant heterogeneity between villages. The lower prevalence levels of epilepsy found in this study could be due to the definition of epilepsy used in this study, increased access to professional support during birth leading to reduced adverse perinatal events or lack of parasites. More than half of all patients had the onset of seizures within the first two decades of life and risk factors include a family history of seizures, problems during delivery, and a history of snoring. HIV was not found to be a risk factor for developing ACE. Given the substantial burden of epilepsy, further research on the burden, including morbidity and mortality, as well as treatment options for and economic burden of epilepsy in rural SSA is warranted.

## Conflicts of Interest

None of the authors has any conflicts of interest to disclose.
